# Video-Based Gait Assessment Using Machine Learning to Classify Age and Sex in Low-Resource Settings: Cross-Sectional Study

**DOI:** 10.2196/76755

**Published:** 2026-03-30

**Authors:** Chanchanok Aramrat, Poppy Alice Carson Mallinson, Papangkorn Inkaew, Pusit Seepheung, Nutchar Wiwatkunupakarn, Nida Buawangpong, Nick Birk, Judith Lieber, Santhi Bhogadi, Hemant Mahajan, Santosh Kumar Banjara, Bharati Kulkarni, Sanjay Kinra, Chaisiri Angkurawaranon

**Affiliations:** 1 Department of Family Medicine Faculty of Medicine Chiang Mai University Chiang Mai Thailand; 2 Global Health and Chronic Conditions Research Group Chiang Mai University Chiang Mai Thailand; 3 Department of Non-Communicable Disease Epidemiology Faculty of Epidemiology and Population Health London School of Hygiene & Tropical Medicine London United Kingdom; 4 Department of Computer Science Faculty of Science Chiang Mai University Chiang Mai Thailand; 5 Harvard TH Chan, School of Public Health Harvard University Cambridge, MA United States; 6 Department of Medical Statistics Faculty of Epidemiology and Population Health London School of Hygiene & Tropical Medicine London United Kingdom; 7 Public Health Foundation of India New Delhi India; 8 National Institute of Nutrition Indian Council of Medical Research Hyderabad, Telangana India

**Keywords:** gait assessment, smartphone video recording, machine learning, age and sex classification, low-resource setting

## Abstract

**Background:**

Gait assessment is an important tool for evaluating health risks in older adults but remains underused in low-resource settings. We explored the feasibility of using a low-cost, simple walking protocol with smartphone video capture to extract health-related gait signals by classifying sex and age. Sex and age are fundamental biological factors linked to most health- and aging-related outcomes. Establishing baseline classification performance provides justification for future exploration of more complex health-related conditions using this protocol.

**Objective:**

This study aimed to assess whether pose parameters derived from smartphone-based gait videos can be used by machine learning models to classify age and sex.

**Methods:**

A cross-sectional study was conducted with 155 participants (Thailand: n=59, 38.1%; India: n=96, 61.9%). Participants performed a simple walking protocol while being recorded using smartphones. Pose estimation was conducted using the MediaPipe algorithm to extract 109 features related to joint distances, angles, and walking speed. For feasibility assessment, we calculated the proportion of recordings for which pose estimation could be extracted. Elastic-net logistic regression and histogram-based gradient boosting classifiers were used for analysis. Model performance was evaluated using 5-fold cross-validation. Outcomes were sex (male vs female) and age group (aged<65 vs ≥65 y).

**Results:**

Pose parameters were successfully extracted from 145 (93.5%) of the 155 video recordings. Among the 145 participants, 94 (64.8%) were female, and 55 (37.9%) were aged 65 years or older. The 2 analytic models demonstrated comparable performance. Sex classification achieved a maximum mean area under the receiver operating characteristic curve of approximately 0.90 (SD 0.06), whereas age classification achieved a maximum mean area under the receiver operating characteristic curve of approximately 0.70 (SD 0.09). Classification performance was primarily influenced by the number of features used, clothing characteristics, and the quality of pose estimation.

**Conclusions:**

This simple smartphone-based gait assessment protocol was able to extract meaningful pose parameters and classify biological features (age and sex). Further studies are warranted to evaluate its potential utility for disease screening, risk stratification, and longitudinal health monitoring.

## Introduction

Older people face substantial unmet health care needs globally [1]. Health problems specific to older people include geriatric syndromes, such as frailty, sarcopenia (muscle loss), and falls, as well as neurodegeneration and multimorbidity [2,3]. These conditions account for substantial loss of quality-adjusted life years as well as economic costs, and their burden is increasing rapidly in the context of population aging [4]. Solutions for scalable geriatric screening and treatment services are urgently needed [5].

The biological processes of aging involve functional decline of various physiological systems, including the musculoskeletal, cardiopulmonary, and neurological systems, impeding the performance of usual functions, such as walking [6]. In geriatric medicine, gait assessment (assessment of how a person walks) is a gold-standard approach for risk-stratifying older patients and screening for a range of health conditions, including risk of falling [7]; frailty; malnutrition [8]; and early symptoms of neurodegenerative disorders, such as dementia [9] or Parkinson disease [10].

Typically, in clinical practice, gait is assessed by clinicians using a battery of simple tests and measurements, which can include walking speed, the Timed Up and Go test, the dynamic gait index, functional ambulation classification, and gait abnormality rating scale [11]. However, gait assessment is generally underused in primary care for assessing aging-related conditions [12]. Barriers to using gait assessment tools include patients’ personal factors (level of education, priority, and physical function), environmental factors (eg, lack of training and facilities, time taken to complete, and cost), and measure-specific factors (eg, subjectivity in gait scoring and lack of recognized population-appropriate tools) [12,13]. In many low-resource settings, this is compounded by a lack of geriatricians and training of primary care physicians in geriatric health issues.

Although technology-driven gait analysis has been widely explored, most studies rely on laboratory settings that require green screens, reflective markers, multiple camera angles, high-resolution recording, and specialized analysis software [14-16]. These requirements make such methods impractical for addressing the diagnostic gap in low-resource or rural health care settings.

A low-cost, automated gait assessment tool could provide a scalable solution to support or replace conventional gait assessments typically performed in clinical practices, particularly in low-resource or rural health care settings [12]. Smartphones are a low-cost and near-ubiquitous technology capable of gathering movement-related information via video recording and sharing this remotely via mobile internet [17]. In health care, machine learning (ML) models applied to high-dimensional data are increasingly capable of assisting or automating clinical diagnoses [18]. Several studies have applied ML to videos acquired in specialized gait laboratories to automatically classify certain diseases and gait abnormalities [19,20]. Smartphones have been used to enhance observational gait analysis among children with cerebral palsy [21]. However, to our knowledge, the potential of using smartphone videos of people walking in free-living environments to automatically characterize aging-related phenotypes, such as healthy aging, frailty, and fall risk, has not been explored.

In this study, we presented a simple walking protocol and an analytic pipeline for extracting gait features from a smartphone video to train ML models to classify aging-related characteristics. We demonstrated proof of concept by using our pipeline to classify the age and sex of participants from 2 distinct geographic settings in India and Thailand. Age and sex are important biological factors linked to most health- and aging-related outcomes. Establishing this baseline performance justified the exploration of more complex health-related conditions using our protocol.

The specific objectives of this study were to (1) establish the feasibility of deriving pose parameters from simple gait assessment protocols based on smartphone videos and (2) assess the ability to differentiate participant age and sex using ML techniques applied to these derived pose estimates.

## Methods

### Study Design, Setting, and Population

A cross-sectional study was conducted in community and primary care sites in India and Thailand, respectively. In India, data were collected in chronic disease screening clinics conducted in 29 villages near Hyderabad. These clinics were part of the fourth follow-up wave (2022-2023) of the Andhra Pradesh Children and Parents’ Study (APCAPS) cohort study and included participants aged 45 years or older, details of which have been described elsewhere [22]. We excluded participants who were unable to walk independently, such as individuals requiring a wheelchair, walking aid, or assistance from another person for locomotion.

In Thailand, participants were recruited from a geriatric clinic at the Faculty of Medicine, Chiang Mai University Hospital, from May 13, 2022, to November 30, 2022. Participants visiting the clinic usually had at least 1 chronic noncommunicable disease (eg, essential hypertension, type 2 diabetes, and dyslipidemia). We invited all patients attending the clinics, with the same exclusion criteria as mentioned in India. Basic demographic data consisting of age, sex, body weight, and height were collected.

### Ethical Considerations

The study protocol was approved by the ethics committees of Indian Council of Medical Research–National Institute of Nutrition (CR/1/V/2023), India; the London School of Hygiene and Tropical Medicine (21771/RR/19113), United Kingdom; and the Research Ethics Committee Panel 5, Faculty of Medicine, Chiang Mai University (study code: FAM-2565-08949), Thailand. All procedures were conducted in accordance with the ethical standards of the respective institutional review boards and the World Medical Association Declaration of Helsinki.

Written informed consent was obtained from all participants before study participation. Participants were informed of their right to withdraw from the study at any time without consequence. All identifying information was removed before analysis, and no personally identifiable information is presented in this publication. Participants received monetary compensation for their participation. In Thailand, participants received 250 Baht (US $6.5).

### Simple Walking Protocols

**Figure 1:**
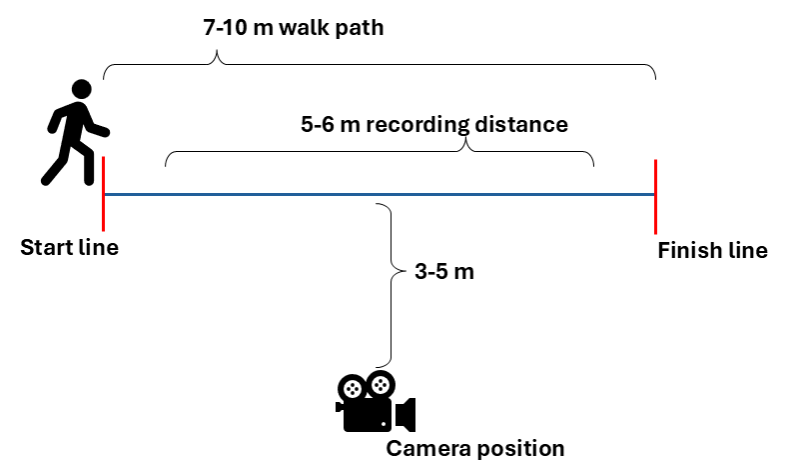
Simple walking protocol.

The simple walking protocol was designed to mirror commonly used clinical gait tests, such as the 10 Meter Walk Test, where patients are timed while walking a distance of 10 m [23], or the Timed Up and Go test, where participants are asked to stand and walk 3 m then turn around and walk back (6 m in total) [24]. For our protocol, patients were asked to walk for 5 to 6 m in a straight line, with a 1- to 2-m walk-in period before recording the videos. The camera was placed 3 to 5 m perpendicular to the midpoint of the walking path. The camera was set between 105 and 130 cm above the ground, such that the whole height of the participant could be captured during the entire 5 to 6 m walking distance (Figure 1). We allowed slight flexibility in the protocol to accommodate different settings where space to conduct this assessment was limited. The detailed protocol and devices used for each site are summarized in Table 1. Participants were allowed to wear any type of clothing without restrictions. Participants were positioned behind the starting line. Video recording was manually initiated immediately before signaling the participant to walk and was maintained for the duration of the walking protocol.

**Table 1:** The protocol and devices used for each site.

	Thailand	India
Walking in distance (m)	7	10
Video recording distance (m)	5	6
Camera height (from the ground; cm)	105	130
Recording devices used	Samsung Galaxy Tab S6 Lite	Samsung Galaxy Tab A7
Resolution	720p	1080p
Frame rate (Hz)	30	30

### Pose Estimation and Gait Cycle Extraction

**Figure 2:**
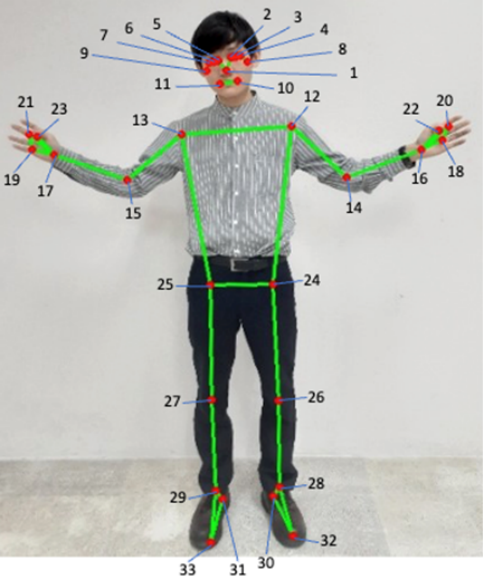
Pose estimates using the MediaPipe algorithm. 1: nose; 2: left eye inner; 3: left eye; 4: left eye outer; 5: right eye inner; 6: right eye; 7: right eye outer; 8: left ear; 9: right ear; 10: mouth left; 11: mouth right; 12: left shoulder; 13: right shoulder; 14: left elbow; 15: right elbow; 16: left wrist; 17: right wrist; 18: left pinky; 19: right pinky; 20: left index; 21: right index; 22: left thumb; 23: right thumb; 24: left hip; 25: right hip; 26: left knee; 27: right knee; 28: left ankle; 29: right ankle; 30: left heel; 31: right heel; 32: left foot index; 33: right foot index.

We applied an open-source pose estimation algorithm, MediaPipe (Google LLC) [25,26], in 2D mode to all video recordings. MediaPipe is an artificial deep neural network algorithm that allows estimates for 33 joints (Figure 2). To identify the gait cycle, we calculated the absolute distance between the left and right ankles. We identified a gait cycle as a time interval of 3 consecutive time points at which the distance between the left and right ankles was 0. In practice, time points at which the distance is exactly 0 are rare due to noise in pose estimations. Therefore, we used time points when the distance was at the extreme local minimum instead of 0. We extracted pose positions only in the second gait cycle, so the pose estimations were not affected by being on the edge (beginning) of the video.

### Feature Extraction

We used the number of frames extracted from the second cycle as a surrogate feature for walking speed. Our method for feature extraction was adopted from a study by Ahmed and Sabir [27]. First, we calculated distances between 15 pairs of joints (Table S1 in Multimedia Appendix 1) in every frame in the second cycle of each participant. For the 15 distance features, we converted all distances in pixels to meters using an in-video reference object placed near the walking path. For participants from Thailand, reference object was a standard measuring tape of 1 m. For participants from India, it was the height of the orange traffic cone used to mark the walking path. Additionally, we extracted 12 different joint angle features (Table S2 in Multimedia Appendix 1) in every frame in the second cycle, as joint angles also provide information regarding health conditions [28-30]. In each participant and for each feature, we calculated the mean, SD, skewness, and kurtosis during the second cycle. This resulted in the extraction of 109 features: 108 joint-derived features and 1 walking speed surrogate feature.

### Data Analysis

Two binary outcomes were examined: (1) sex (male vs female) and (2) age group (<65 vs ≥65 y). Given the high number of features, model feasibility was explored by systematically varying the number of input features rather than fixing a single feature set. Before model training, the number of features to be used was prespecified and iteratively varied from 1 to 50. For each predefined feature subset size, the complete model training and evaluation pipeline was repeated independently.

### Evaluation Strategies

**Figure 3:**
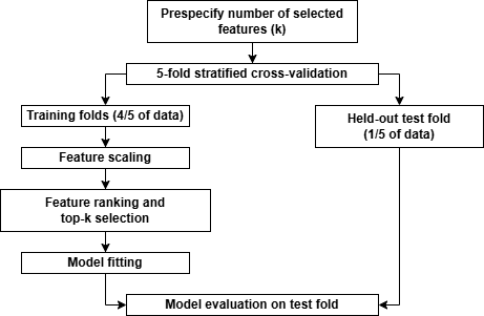
Evaluation strategies.

As part of the feasibility assessment, we calculated the proportion of recordings for which pose estimation could be extracted. Model performance was evaluated using a repeated stratified cross-validation framework. Specifically, 5-fold cross-validation was used, with folds stratified by the outcome to preserve class proportions. This procedure was repeated 20 times using different random splits, yielding 100 resampled train-test evaluations for each feature subset size (Figure 3).

### Feature Scaling, Ranking, and Selection

All data preprocessing and modeling steps—including feature scaling, feature ranking and selection, model fitting, and probability estimation—were performed entirely within each training fold and then applied to the corresponding held-out test fold. This strict separation was enforced to prevent information leakage between training and evaluation phases.

Within each training fold, continuous features were standardized using z score normalization based on the training data only. Feature ranking was then performed using an elastic net–regularized logistic regression (LR) model fitted to the scaled training data. The elastic net penalty combined L1 and L2 regularization (hyperparameter details are provided in Table S3 in Multimedia Appendix 1). To account for class imbalance during feature ranking, balanced class weights were applied.

Features were ranked according to the absolute magnitude of their fitted regression coefficients. For each cross-validation fold and repetition, the top-ranked features corresponding to the predefined feature subset size were selected. Feature ranking and selection were recomputed independently for every fold and repetition.

### Statistical Analysis and Evaluation Metrics

Two classification models were evaluated: (1) elastic-net LR, a linear classifier, was used as the baseline model for feasibility assessment and (2) a histogram-based gradient boosting (HistGB) classifier, a tree-based ensemble method, was used to probe potential nonlinear relationships in the data.

Both models were trained using only the selected features within each training fold. Balanced class weights (or sample weights, where applicable) were used to mitigate class imbalance. Hyperparameter specifications for both models are reported in Table S3 in Multimedia Appendix 1.

As an assessment of the acceptability of the simple gait protocol to derive biological features (age and sex), model discrimination performance was assessed using the area under the receiver operating characteristic curve (AUC-ROC), overall accuracy, per-class sensitivity, per-class *F*_1_-score, and Matthews correlation coefficient (MCC).

### Explore Factors Affecting Classification

To explore factors associated with classification, we conducted 5 sensitivity analyses.

First, classification performance estimates were aggregated across folds and repetitions to generate performance curves versus the number of features, allowing assessment of classification feasibility as a function of feature dimensionality.

Second, to explore how body shape may influence the classification performance, we stratified the performance metrics by BMI 25 kg/m^2^ or greater and less than 25 kg/m^2^, which served as a rough surrogate for body shape.

Third, we repeated the analysis by site subgroups (India and Thailand) to explore whether the pooling of the dataset from the 2 sites diluted the meaningful signals within, exploring the generalizability of the data collection protocol.

Fourth, we identified the 10 individuals for whom the models demonstrated good and poor performance. We qualitatively reviewed the walking videos and documented any factors that may contribute to poor pose estimation, such as clothing, environment, the alignment of the camera, and the quality of pose estimation.

Fifth, we plotted the distribution of the commonly selected features by outcomes to investigate whether the distribution of features differed by outcomes.

## Results

### Overview

A total of 155 videos were recorded (Thailand: n=59, 38.1%; India: n=96, 61.9%). Upon quality assessment and applying the pose estimation algorithm, we were able to extract pose parameters from 145 (93.5%) videos. In the remaining 10 (6.5%) videos, pose estimation failed due to incorrect joint estimations, often capturing nonhuman elements instead. In 1 case, there was an object that obstructed the participant’s image. Of the 145 participants, 51 (35.2%) were from Thailand, and 94 (64.8%) were from India. In total, 94 (64.7%) participants were female, and 55 (37.9%) participants were aged 65 years or older (Table 2).

**Table 2:** Participant demographics.

	Total (N=145)	Thailand (n=51)	India (n=94)
Female, n (%)	94 (64.8)	33 (64.7)	61 (64.9)
Age, median (IQR)	50 (48-69)	64 (29-67)	50 (49-70)
Aged ≥65 y, n (%)	55 (37.9)	22 (43.1)	33 (35.1)
BMI, median (IQR)	22.96 (19.84-26.40)	23.26 (20.13-27.58)	22.70 (19.73-25.13)
BMI ≥25, n (%)	51 (35.2)	21 (41.2)	30 (31.9)

### Performance for Sex Classification

**Figure 4:**
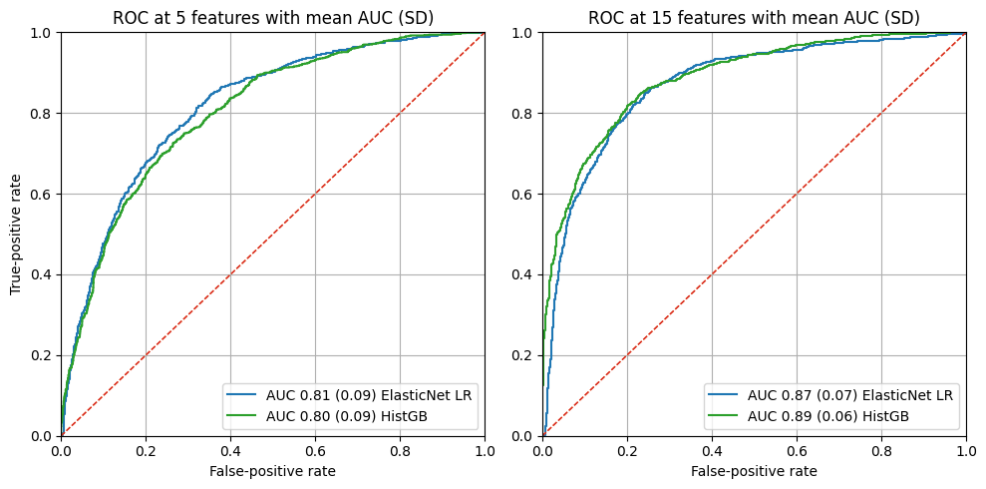
Performance for sex classification. AUC: area under the curve; HistGB: histogram-based gradient boosting; LR: logistic regression; ROC: receiver operating characteristic.

Using 5 features, both models demonstrated good discriminative ability, achieving area under the curve (AUC) values above 0.8. Increasing the number of selected features to 15 further improved discrimination, with AUC values approaching 0.9 for both models (Figure 4).

Detailed results for additional evaluation metrics of sex classification across all feature subset sizes are presented in Figure S1 in Multimedia Appendix 1.

### Performance for Age Classification

**Figure 5:**
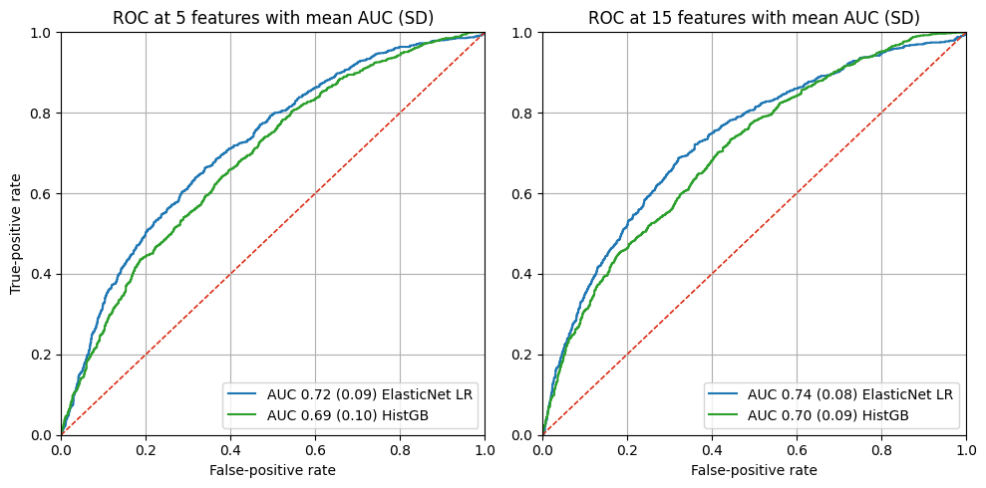
Performance for age classification. AUC: area under the curve; HistGB: histogram-based gradient boosting; LR: logistic regression; ROC: receiver operating characteristic.

Using 5 selected features, both models demonstrated modest discriminative performance. ElasticNet LR achieved a mean AUC of 0.72 (SD 0.09), while HistGB achieved a mean AUC of 0.69 (SD 0.10). Increasing the number of selected features to 15 resulted in a small but consistent improvement in discrimination, with mean AUC values increasing to 0.74 (SD 0.08) for ElasticNet LR and 0.70 (SD 0.09) for HistGB (Figure 5).

Detailed results for all evaluation metrics across feature subset sizes are presented in Figure S1 in Multimedia Appendix 1.

### Exploration of Factors Affecting Classification

#### Number of Features

Performance metrics stratified by the number of features used suggested a strong influence of the number of features in sex classification when fewer than 10 features were used but only had a weak influence on age group classification (Figure S1 in Multimedia Appendix 1). Across the full range of feature subset sizes (top-k=1-50), AUC-ROC increased rapidly from approximately 0.6 with a single feature to nearly 0.9 at approximately 10 features, after which performance plateaued. HistGB consistently achieved slightly higher AUC-ROC values than ElasticNet LR, although the variability across resampling iterations overlapped. Other evaluation metrics—including accuracy, sensitivity, *F*_1_-score, and MCC—exhibited similar patterns, with steep improvements at low feature counts and minimal gains beyond approximately 8 to 10 features. Sensitivity and *F*_1_-scores were consistently higher for female classification than for male classification across both models. Overall, these metrics demonstrated convincing discriminative performance for feasibility evaluation (Figure S1 in Multimedia Appendix 1).

In contrast, for age classification, across the full range of feature subset sizes (top-k=1-50), AUC-ROC increased gradually from approximately 0.65 to 0.70 at low feature counts and plateaued after approximately 10 to 15 features at an AUC-ROC of 0.70 to 0.75, with only minimal gains thereafter (Figure S1 in Multimedia Appendix 1). ElasticNet LR consistently achieved slightly higher AUC-ROC values than HistGB across most feature levels, although the performance variability across resampling iterations overlapped. Other evaluation metrics exhibited similar patterns. Accuracy improved modestly with increasing feature counts and stabilized beyond approximately 10 features. Sensitivity and *F*_1_-scores were consistently higher for younger adults than for older adults across both models, while performance for older adults remained lower and more variable. MCC values increased slightly at low feature counts but remained relatively low overall. The metrics reflected the limited discriminative separation between age groups (Figure S1 in Multimedia Appendix 1).

#### BMI Measurements

In both sex and age classification, the graphs generally showed slight separation of performance metrics between the 2 groups (Figures S2 and S3 in Multimedia Appendix 1). The models tended to perform slightly better for individuals with higher BMI (>25 kg/m^2^) for age-sex classification but lower in age classification. Revealing that there is the possibility that the shape of a person may have an effect on modifying, although to a small degree.

#### Variability in Data Collection Between Sites

Rerunning the model training and evaluation separately by site (Thailand and India; Figures S4-S7 in Multimedia Appendix 1), the performance metrics in the Thailand dataset showed a similar pattern when compared to the pooled dataset in both classes. AUC-ROC for sex classification increased in the first 5 features, then plateaued at about 10 features at a value just under 0.90 in both analysis models. While in age classification, the AUC-ROC slightly increased in the first 5 features, then plateaued at about 10 features as AUC-ROC of just under 0.70. These patterns were observed in other performance metrics as well (Figures S4 and S6 in Multimedia Appendix 1).

For the India dataset, for sex classification, similar patterns compared to the pooled dataset were found; however, at the plateau, the performance reached slightly higher compared to the pooled dataset at approximately AUC-ROC of 0.90 in ElasticNet LR and 0.95 in the HistGB model. For age classification, the prediction performance started low at an AUC-ROC of approximately 0.60 to 0.65, then gradually increased to more than 0.75 at 50 features. These patterns were observed in other performance metrics from the India dataset as well (Figures S5 and S7 in Multimedia Appendix 1).

#### Qualitative Exploration

For sex prediction, the 5 individuals with the worst prediction for sex were all male, while the top 5 were all female. For age prediction, a similar trend emerged in that the 10 best predictions occurred in individuals younger than 65 years (and they were all female). In comparison, 9 of the 10 worst predictions occurred in individuals aged 65 years and older (5 male and 5 female). A qualitative review of videos revealed that among those for whom the model performed poorly, there were some jitters in the pose estimations among those with male skirts or trousers (Tables S4 and S5 in Multimedia Appendix 1).

#### Distribution of Commonly Selected Features

Exploring the distribution of the commonly selected features demonstrated that for sex discrimination, the distribution of many of the selected features varied by sex (Figure S8 in Multimedia Appendix 1). However, for age discrimination, there was a more substantial overlap in the distribution of the features commonly selected (Figure S9 in Multimedia Appendix 1).

## Discussion

### Principal Findings

In this proof-of-concept paper, we demonstrated the feasibility of a simple standardized walking protocol. Our method enabled us to estimate poses and extract features from more than 93.5% (145/155) of the videos analyzed. Moreover, we demonstrated that there are signals that allow simple ML models to discriminate sex and age, important biological factors linked to most health- and aging-related outcomes, making a case for the exploration of more complex health-related phenotypes, building on the walk protocol and analytic pipeline in the future.

All classifiers produced by the proposed framework achieved a mean AUC-ROC of approximately 0.80 to 0.90 for sex prediction. Our model performance was similar to that of another study, which used inertial sensors (accelerometers and gyro sensors) to classify sex [31,32]. However, our method did not perform as well when compared to those using ultrawide band radar [33] or Kinect sensors [34], which reported accuracies exceeding 90%. It is likely that Kinect sensors and radar can more effectively capture gait parameters. Kinect sensors use at least 2 sensors (red, green, blue image and depth sensors) to provide information on joint positions [34], and radar sensors provide information on surface geometry and orientation of an individual [33], while inertial sensors only capture the body’s acceleration and rotation data [31,32]. Any improvements in data collection or pose estimation—such as higher resolution cameras or enhanced pose estimation algorithms—could potentially improve the accuracy and stability of gait parameter measurements, thereby enhancing sex classification. This assumption is further supported by our observation that among individuals in whom the model performed poorly, significant joint position jitters were observed.

For age prediction, our method achieved a mean AUC-ROC of approximately 0.65 to 0.70. Another similar study that was conducted in 48 female individuals who were classified as young (aged 21-30 y) and older (aged 55-70 y) adults using a support vector machine–based model achieved an accuracy of more than 95% [35]. While our population differs from their dataset, which may limit direct comparability, their pose estimation data were captured using reflective markers and 8 synchronized digital infrared high-speed cameras. This setup likely produced higher-quality pose estimation compared to our method. This highlights that improving pose estimation quality could significantly enhance age prediction accuracy. Another possible explanation is that the relatively similar ages within our study population made it more challenging to discriminate between age groups.

Stratification of evaluation metrics by BMI demonstrated slight separation between individuals with high and low BMI. This suggests that BMI, or to an extent, body size and shape, should be accounted for to assess the true gait pattern. Qualitative assessment of walking videos suggests that, among the individuals in whom the ML model performed poorly, it was observed that all were wearing loose-fitting clothes. Thus, to improve the accuracy of pose estimates, we suggest that individuals should not wear very loose-fitting clothes for accurate pose estimation [36,37]. The distribution of the extracted features also overlapped, particularly for our age prediction model. This suggests that the other transformations could be applied to some features. They may improve the ability to discriminate biological factors (ie, age and sex) of the features. Additionally, ML models of time-series analysis, such as recurrent neural networks and long short-term memory networks, could be applied instead of the traditional model used in this study. However, those ML models would require large datasets and should be the objective of future studies.

### Strengths and Limitations

There are strengths and limitations to our study. The main strength of this work is the ability to demonstrate the feasibility of using a simple and low-cost walking protocol using smartphone cameras to extract meaningful features in 2 diverse real-world settings with participants wearing typical clothes in primary care from India and Thailand. This very simple protocol could be implemented in a range of community settings or without specialist input. This study explored variations in performance between the 2 different settings, Thailand and India, which represented different ethnicities, general body shape, and clothing and were slightly different in the walking protocol. When stratified by site, both sites achieved similar or only slightly better discrimination metrics, which represents the robustness of our methods across diverse settings.

There were some limitations due to the small sample size. As demonstrated in the model performance analysis, it seems that the class imbalance problem occurred. Individuals from the majority class in the data are predicted more accurately than individuals from the minority class. Despite this, the models demonstrated reasonable discriminatory capability and did not merely default to predicting the more prevalent classes. The problem could be resolved with a more balanced and larger dataset in future work, which could also allow for different resampling methods to address this issue [38]. With a larger sample size, it is possible that the discriminatory performance could be improved, and other time-series analyses can be explored for better extraction of health-related signals [39].

Our feature extraction approach may be subject to measurement noise. Gait speed was estimated from the number of frames within a single gait cycle, which may not fully capture true walking speed. In addition, all features were derived from only 1 gait cycle. Given the inherent jitter and variability in pose estimation, reliance on a single cycle may introduce noise and reduce the stability of the extracted features, thereby affecting model performance.

Several aspects of the proposed video-based gait assessment protocol require further refinement to enhance signal quality and analytic performance. First, improvements can be made at the level of raw data acquisition by optimizing the walking protocol. This includes providing clearer guidance on clothing to avoid occlusion of key anatomical landmarks as well as exploring the use of alternative or multiview recording configurations, either in addition to or instead of a perpendicular view, to improve the robustness and accuracy of pose estimation.

### Implications for Future Developments to Improve Performance in Clinical Applications

Signal detection and analytic methods warrant further development. Future work should examine the use of multiple gait cycles rather than restricting analysis to a single cycle to better capture within-person variability and temporal dynamics. In addition, comparative evaluation of different pose-estimation algorithms and feature-extraction strategies may help identify representations that are more sensitive to clinically relevant gait characteristics. Finally, while this study focused on relatively simple classification models to assess feasibility, more complex modeling approaches—such as deep neural networks, graph convolutional networks, or convolutional neural network–based architectures—may further improve performance as larger and more diverse datasets become available.

### Conclusions

This simple smartphone-based gait assessment protocol was able to extract meaningful pose parameters and classify biological features (age and sex) and showed promising robustness for deployment in diverse real-world outpatient settings. Although these findings support its feasibility, several aspects of the walking protocol and analytic methods warrant further refinement, including optimization of data capture and feature extraction. Future research should evaluate the utility of this approach for disease screening, risk stratification, and longitudinal monitoring across broader clinical populations.

## Appendix

Multimedia Appendix 1 Analyses of sex and age classification performance, feature characteristics, model hyperparameter specifications, and qualitative assessments across Thailand and India datasets.

## Abbreviations

APCAPS: Andhra Pradesh Children and Parents’ Study
AUC: area under the curve
AUC-ROC: area under the receiver operating characteristic curve
HistGB: histogram-based gradient boosting
MCC: Matthews correlation coefficient
ML: machine learning

## Data Availability

The data supporting the findings of this study are not publicly available due to privacy concerns, as they include identifiable walking videos of study participants, but deidentified data from Thailand are available from the corresponding author on reasonable request, subject to ethical approval and data-sharing agreements. Deidentified data from the APCAPS cohort are made available for sharing with bona fide collaborating researchers (subject to the APCAPS terms and conditions for collaboration, which are designed to ensure compatibility with original study ethics and participant consents). Applications can be made by submission of a completed APCAPS Collaborator Request Form [40] to apcaps.crf@gmail.com.

## References

[1] Kowal P, Corso B, Anindya K, Andrade FC, Giang TL, Guitierrez MT, Pothisiri W Working paper: prevalence of unmet health care need in older adults in 83 countries – measuring progressing towards universal health coverage in the context of global population ageing. World Health Organization.

[2] Chan AK, Tamrakar M, Jiang CM, Lo EC, Leung KC, Chu C (2021). Common medical and dental problems of older adults: a narrative review. Geriatrics (Basel).

[3] Abdi S, Spann A, Borilovic J, de Witte L, Hawley M (2019). Understanding the care and support needs of older people: a scoping review and categorisation using the WHO international classification of functioning, disability and health framework (ICF). BMC Geriatr.

[4] Fulmer T, Reuben DB, Auerbach J, Fick DM, Galambos C, Johnson KS (2021). Actualizing better health and health care for older adults. Health Aff (Millwood).

[5] Mahmoud A, Goodwin VA, Morley N, Whitney J, Lamb SE, Lyndon H, Creanor S, Frost J, DREAM Study Team (2024). How can we improve comprehensive geriatric assessment for older people living with frailty in primary care and community settings? A qualitative study. BMJ Open.

[6] Gamwell HE, Wait SO, Royster JT, Ritch BL, Powell SC, Skinner JW (2022). Aging and gait function: examination of multiple factors that influence gait variability. Gerontol Geriatr Med.

[7] Cardon-Verbecq C, Loustau M, Guitard E, Bonduelle M, Delahaye E, Koskas P, Raynaud-Simon A (2017). Predicting falls with the cognitive timed up-and-go dual task in frail older patients. Ann Phys Rehabil Med.

[8] Bortone I, Sardone R, Lampignano L, Castellana F, Zupo R, Lozupone M, Moretti B, Giannelli G, Panza F (2021). How gait influences frailty models and health-related outcomes in clinical-based and population-based studies: a systematic review. J Cachexia Sarcopenia Muscle.

[9] Mc Ardle R, Del Din S, Donaghy P, Galna B, Thomas AJ, Rochester L (2021). The impact of environment on gait assessment: considerations from real-world gait analysis in dementia subtypes. Sensors (Basel).

[10] Leddy AL, Crowner BE, Earhart GM (2011). Functional gait assessment and balance evaluation system test: reliability, validity, sensitivity, and specificity for identifying individuals with Parkinson disease who fall. Phys Ther.

[11] Mohamed O, Appling H, Chui KK, Jorge M, Yen SC, Lusardi MM (2020). Clinical assessment of gait. Orthotics and Prosthetics in Rehabilitation 4th ed.

[12] Hulleck AA, Menoth Mohan D, Abdallah N, El Rich M, Khalaf K (2022). Present and future of gait assessment in clinical practice: towards the application of novel trends and technologies. Front Med Technol.

[13] Jang HY, Kim YL, Oh JL, Lee SM (2017). Barriers to using balance and gait assessment tools by physical therapists in patients with neurological impairments: a systematic review. J Clin Res Bioeth.

[14] Fernandez KM, Roemmich RT, Stegemöller EL, Amano S, Thompson A, Okun MS, Hass CJ (2013). Gait initiation impairments in both essential tremor and Parkinson's disease. Gait Posture.

[15] Moon G, Cho J, Choi H, Kim Y, Kim GD, Jang SH (2025). AI-based severity classification of dementia using gait analysis. Sensors (Basel).

[16] Alizadeh Noghani M, Green S, Bolívar-Nieto E (2025). Whole-body optical marker and ground reaction force data of healthy humans performing non-cyclic activities. Sci Data.

[17] Su D, Liu Z, Jiang X, Zhang F, Yu W, Ma H, Wang C, Wang Z, Wang X, Hu W, Manor B, Feng T, Zhou J (2021). Simple smartphone-based assessment of gait characteristics in Parkinson disease: validation study. JMIR Mhealth Uhealth.

[18] Harris EJ, Khoo IH, Demircan E (2021). A survey of human gait-based artificial intelligence applications. Front Robot AI.

[19] Wahid F, Begg RK, Hass CJ, Halgamuge S, Ackland DC (2015). Classification of Parkinson's disease gait using spatial-temporal gait features. IEEE J Biomed Health Inform.

[20] Kidziński Ł, Yang B, Hicks JL, Rajagopal A, Delp SL, Schwartz MH (2020). Deep neural networks enable quantitative movement analysis using single-camera videos. Nat Commun.

[21] Kephart DT, Laing SR, Bagley A, Davids JR, Kulkarni VA (2020). Gait analysis at your fingertips: accuracy and reliability of mobile app enhanced observational gait analysis in children with cerebral palsy. J Pediatr Orthop Soc North Am.

[22] Lieber J, Banjara SK, Mallinson PA, Mahajan H, Bhogadi S, Addanki S, Birk N, Song W, Shah AS, Kurmi O, Iyer G, Kamalakannan S, Kishore Galla R, Sadanand S, Dasi T, Kulkarni B, Kinra S (2023). Burden, determinants, consequences and care of multimorbidity in rural and urbanising Telangana, India: protocol for a mixed-methods study within the APCAPS cohort. BMJ Open.

[23] Watson MJ (2002). Refining the ten-metre walking test for use with neurologically impaired people. Physiotherapy.

[24] Browne W, Nair BR (2019). The timed up and go test. Med J Aust.

[25] MediaPipe Pose landmark detection guide. GoogleAI.

[26] Bazarevsky V, Grishchenko I, Raveendran K, Zhu T, Zhang F, Grundmann M BlazePose: on-device real-time body pose tracking. arXiv.

[27] Ahmed MH, Sabir AT (2017). Human gender classification based on gait features using Kinect sensor. Proceedings of the 3rd IEEE International Conference on Cybernetics.

[28] Honda K, Sekiguchi Y, Izumi SI (2023). Effect of aging on the trunk and lower limb kinematics during gait on a compliant surface in healthy individuals. Biomechanics.

[29] Zanardi AP, da Silva ES, Costa RR, Passos-Monteiro E, Dos Santos IO, Kruel LF, Peyré-Tartaruga LA (2021). Gait parameters of Parkinson's disease compared with healthy controls: a systematic review and meta-analysis. Sci Rep.

[30] Zhong Q, Ali N, Gao Y, Wu H, Wu X, Sun C, Ma J, Thabane L, Xiao M, Zhou Q, Shen Y, Wang T, Zhu Y (2021). Gait kinematic and kinetic characteristics of older adults with mild cognitive impairment and subjective cognitive decline: a cross-sectional study. Front Aging Neurosci.

[31] Gillani SI, Azam MA, Ehatisham-ul-Haq M (2020). Age estimation and gender classification based on human gait analysis. Proceedings of 2020 International Conference on Emerging Trends in Smart Technologies.

[32] Khabir KM, Siraj MS, Ahmed M, Ahmed MU (2019). Prediction of gender and age from inertial sensor-based gait dataset. Proceedings of the Joint 8th International Conference on Informatics, Electronics & Vision and 3rd International Conference on Imaging, Vision & Pattern Recognition.

[33] Saleem AA, Siddiqui HU, Sehar R, Dudley S (2024). Gender classification based on gait analysis using ultrawide band radar augmented with artificial intelligence. Expert Syst Appl.

[34] Azhar M, Ullah S, Ullah K, Syed I, Choi J (2022). A gait-based real-time gender classification system using whole body joints. Sensors (Basel).

[35] Eskofier BM, Federolf P, Kugler PF, Nigg BM (2013). Marker-based classification of young-elderly gait pattern differences via direct PCA feature extraction and SVMs. Comput Methods Biomech Biomed Engin.

[36] Matsumoto T, Shimosato K, Maeda T, Murakami T, Murakoso K, Mino K, Ukita N (2020). Human pose annotation using a motion capture system for loose-fitting clothes. IEICE Trans Inf Syst.

[37] Yamaguchi T, Mikami D, Matsumura S, Saijo N, Kashino M Pose estimation for human wearing loose-fitting clothes: obtaining ground truth posture using HFR camera and blinking LEDs. arXiv.

[38] Sowjanya AM, Mrudula O (2023). Effective treatment of imbalanced datasets in health care using modified SMOTE coupled with stacked deep learning algorithms. Appl Nanosci.

[39] Zhang S, Wang Y, Li A (2019). Gait-based age estimation with deep convolutional neural network. Proceedings of the 2019 International Conference on Biometrics.

[40] APCAPS and London School of Hygiene & Tropical Medicine (LSHTM).

