# Supplementary material for: Video-Based Gait Assessment Using Machine Learning to Classify Age and Sex in Low-Resource Settings: Cross-Sectional Study
Source: JMIR Form Res. 2026 Mar 30;10:e76755. doi: 10.2196/76755 (PMC13077277; doi:10.2196/76755)
Supplement: Multimedia Appendix 1 [file formative_v10i1e76755_app1.docx]

**Figure S1**: Sex and age classification performance metrics vs number of features, combined dataset (Thailand and India)


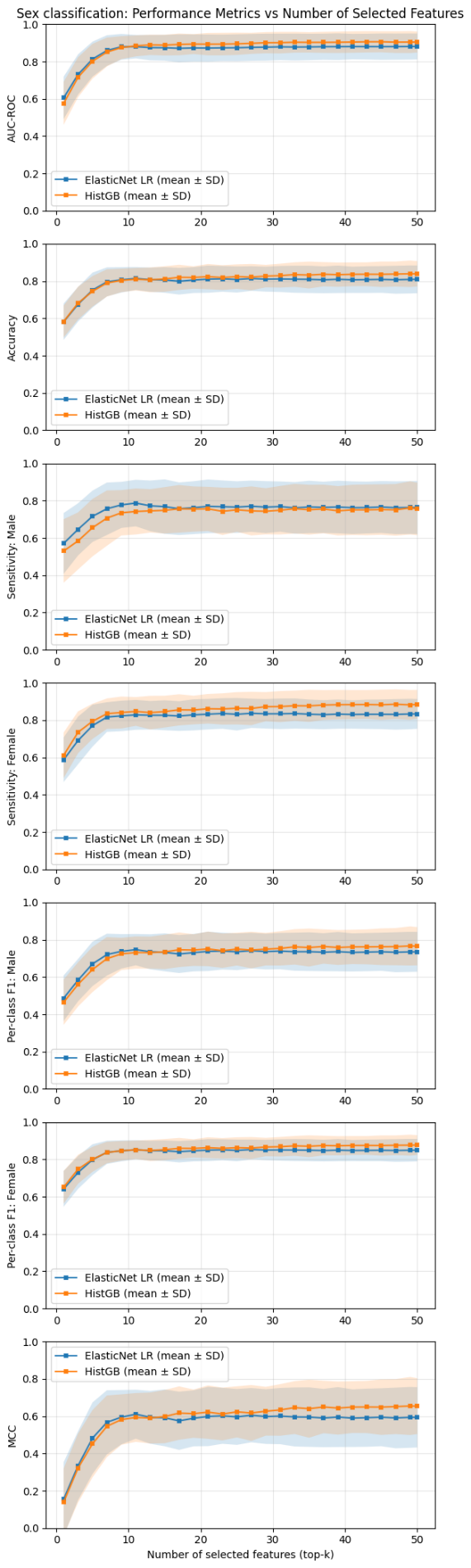

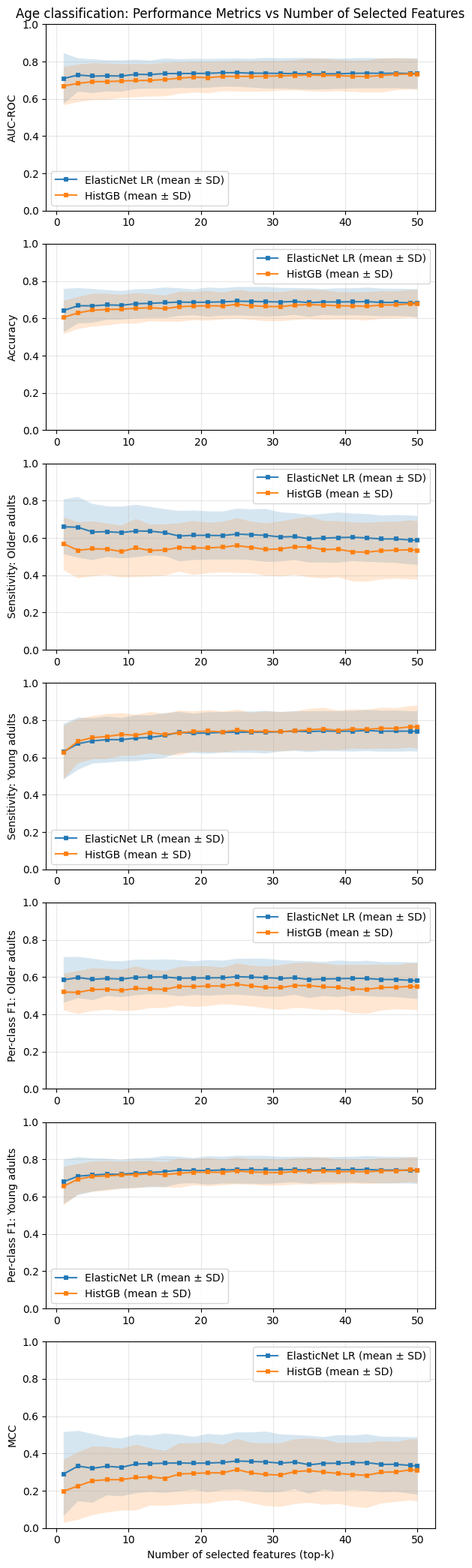


**Figure S2:** Sex classification, stratified by BMI groups


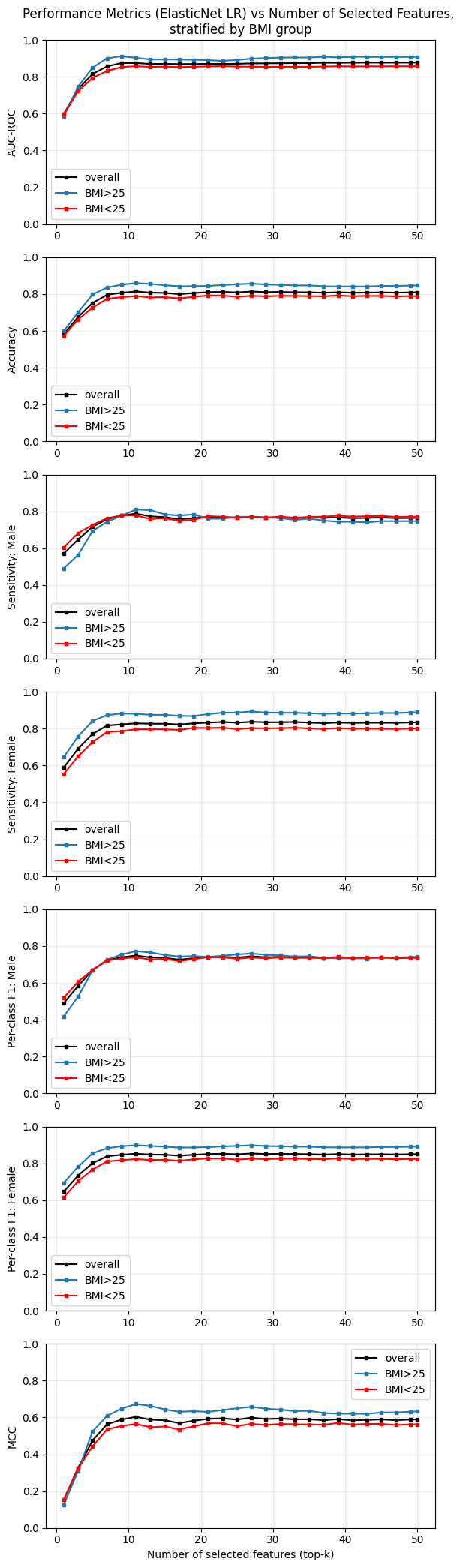

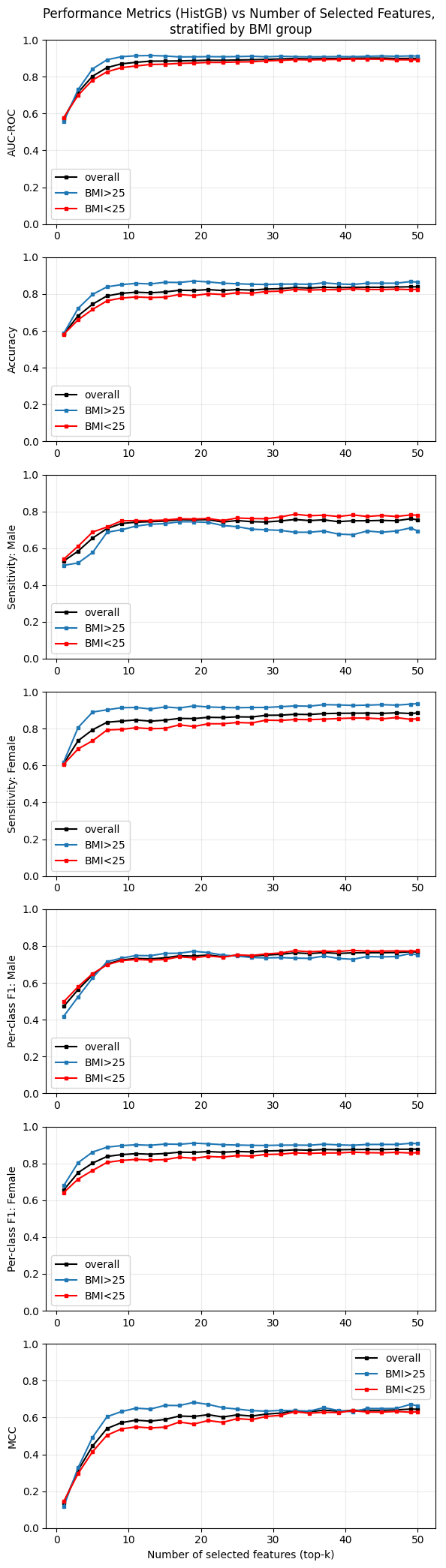


**Figure S3:** Age classification, stratified by BMI groups


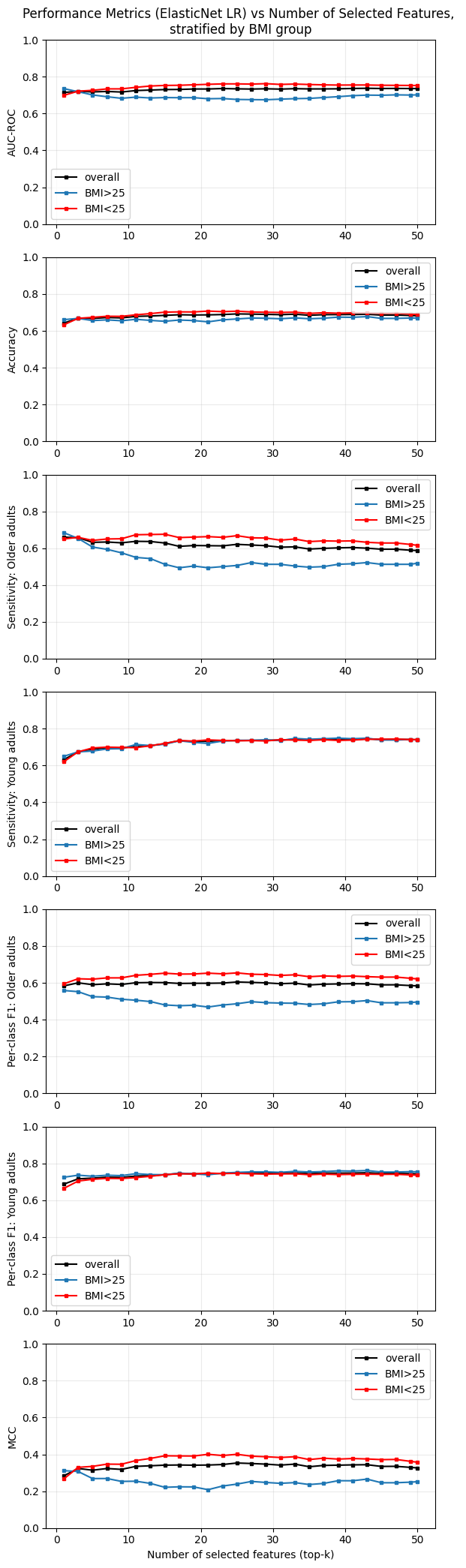

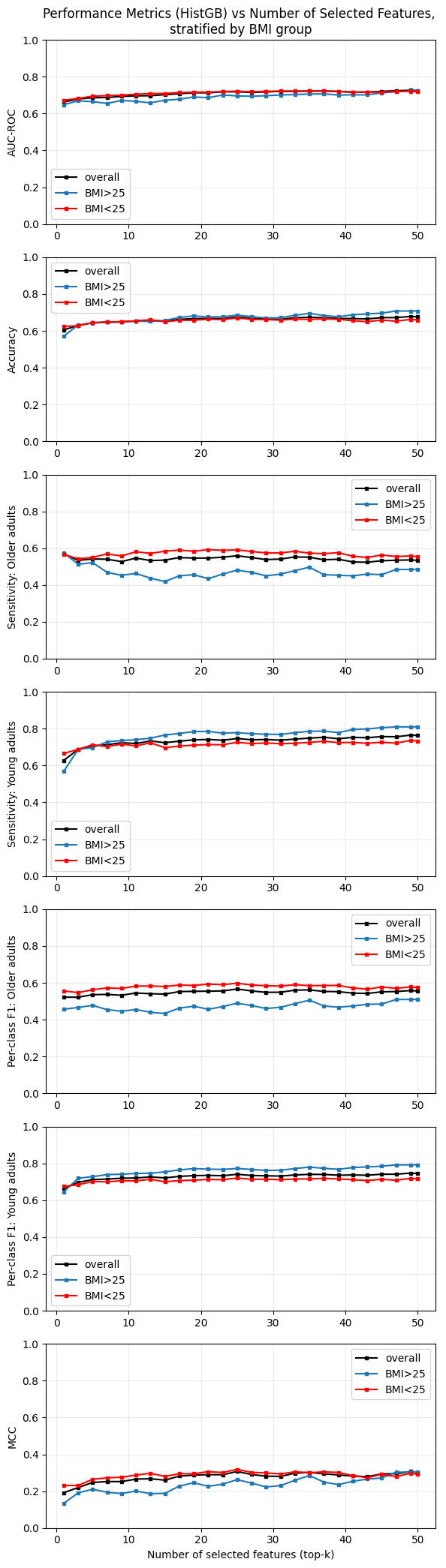


**Figure S4:** Sex and age classification ROC at 5 features and 15 features, Thailand dataset.


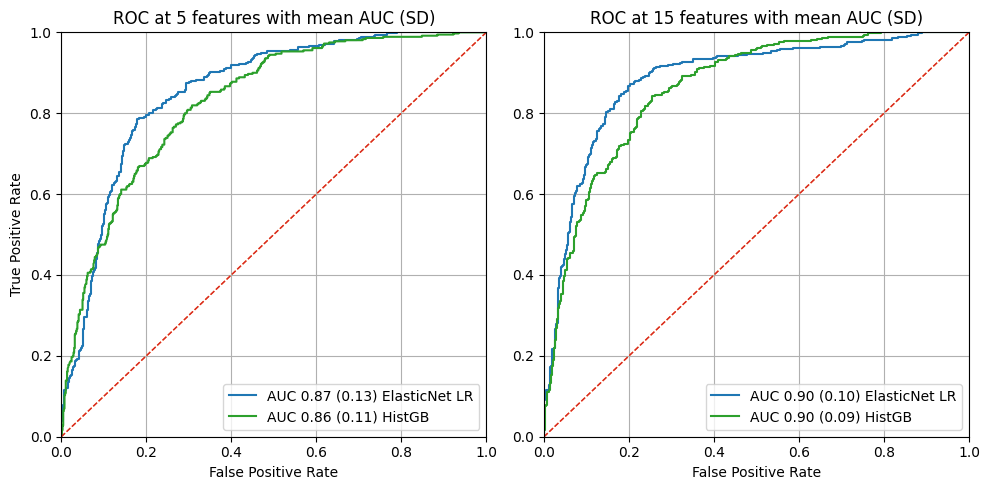

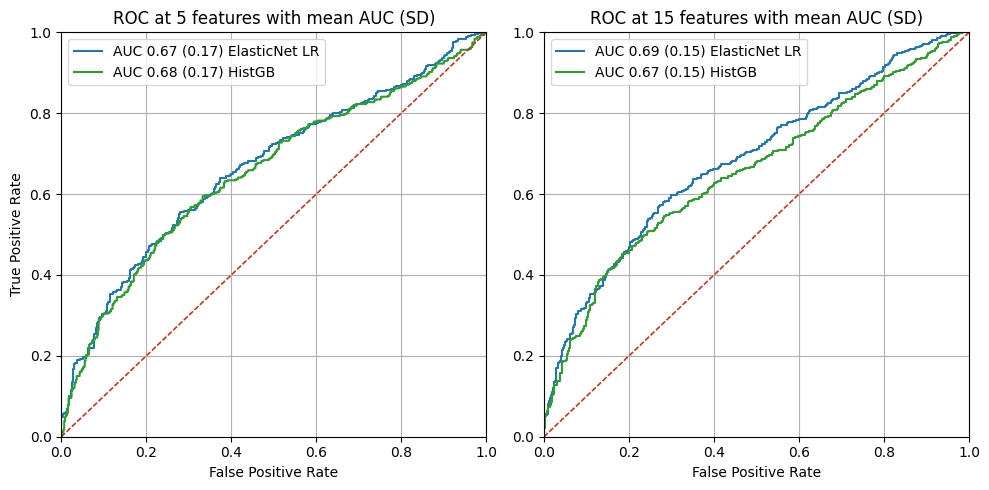


**Figure S5:** Sex and age classification ROC at 5 features and 15 features, India dataset.


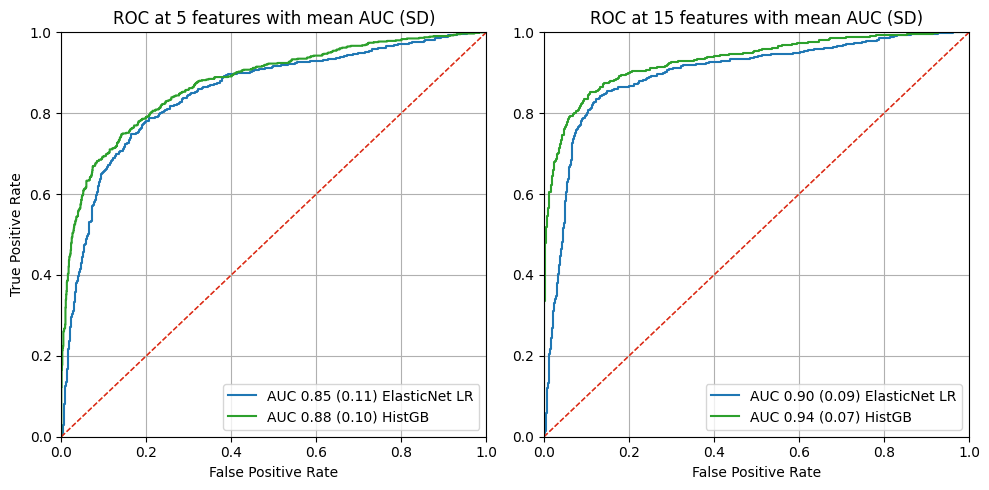

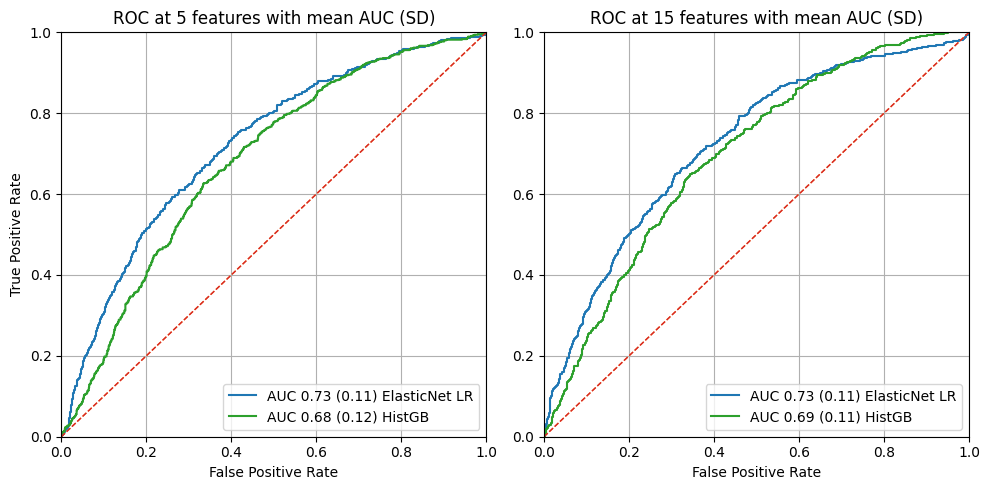


**Figure S6**: Age and sex classification performance metrics vs number of features, Thailand dataset.


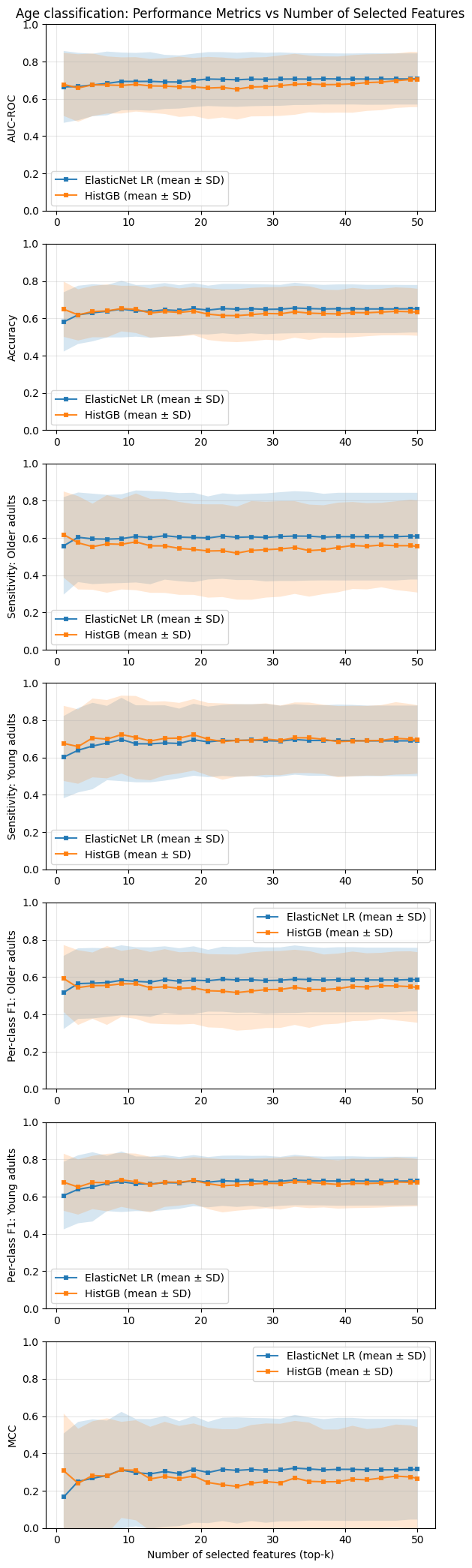

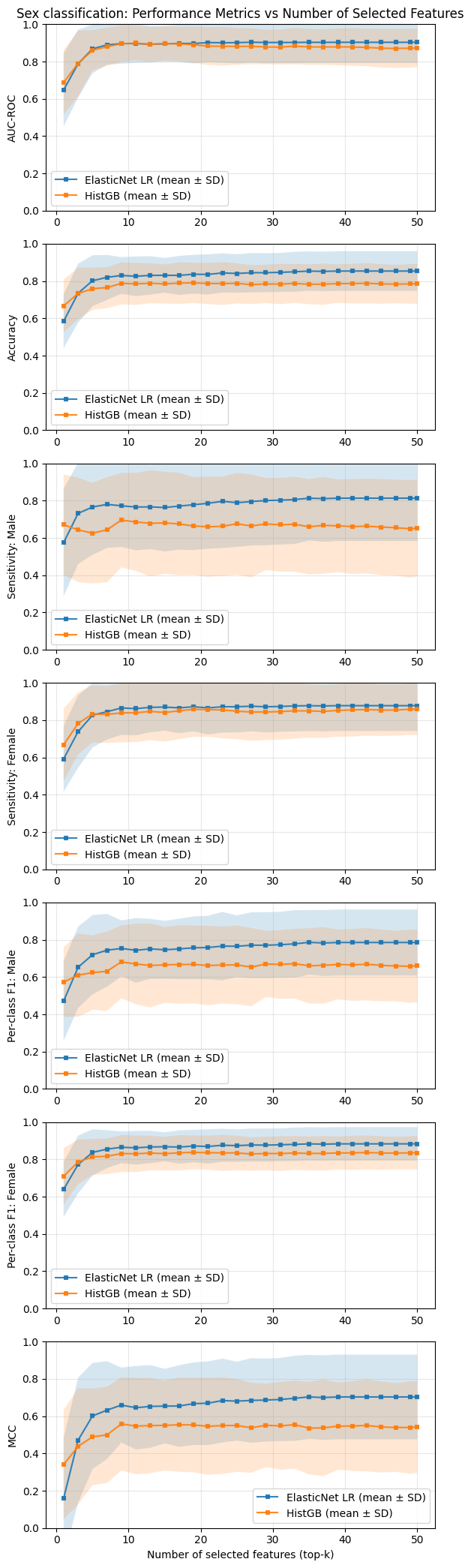


**Figure S7:** Age and sex classification performance metrics vs number of features, India dataset.


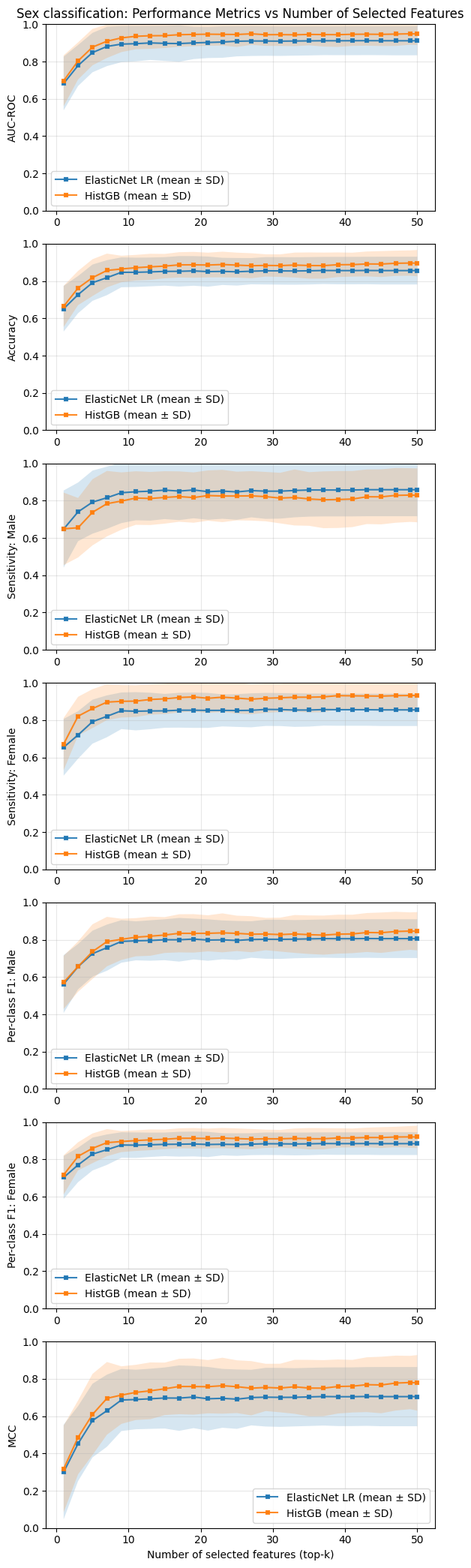

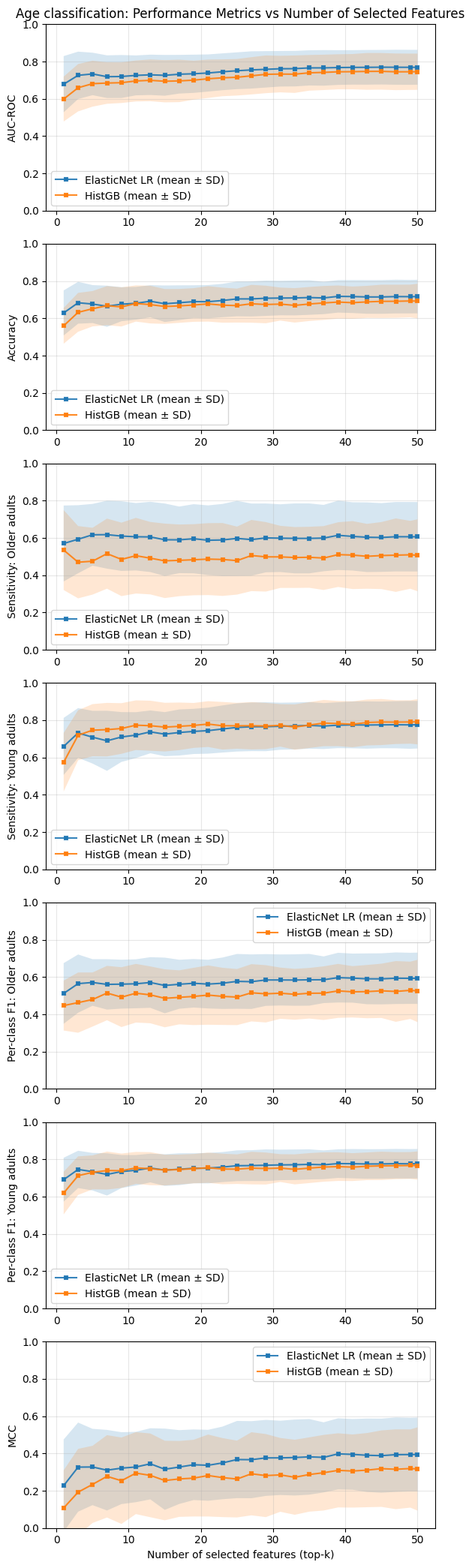


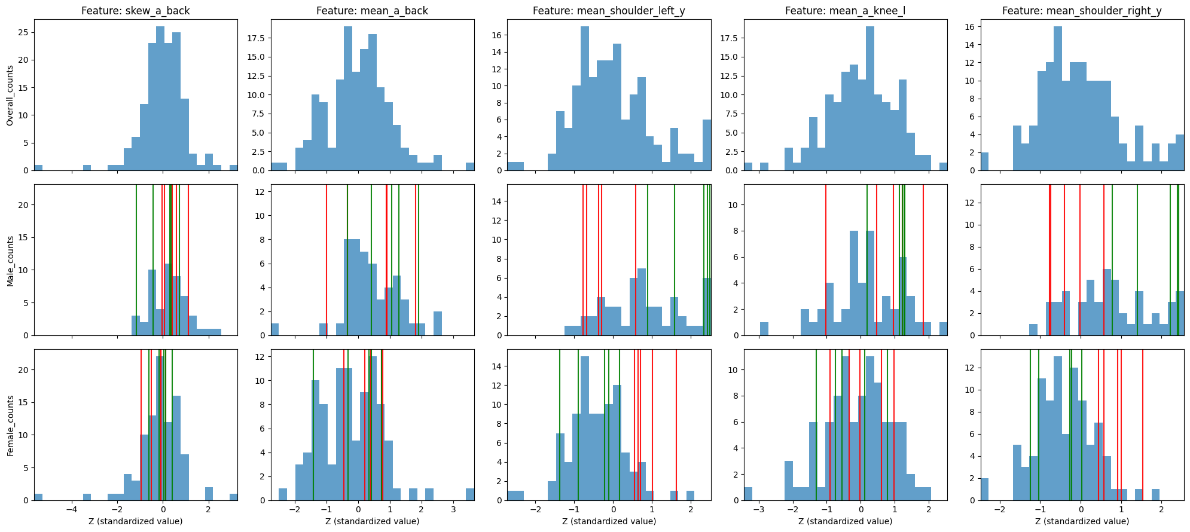
 **Figure S8:** Distribution of features frequently selected for sex discrimination.

**|** Cases where models performed well **|** Cases where models did not perform well

Figure S8 Legend: In each feature, the top row represents the overall distribution, the middle row represents the distribution in female, bottom row represents the distribution in male. The green vertical dash line represents 5 cases that were well classified by the models in each sex, while red represents 5 cases where they were poorly classified within each sex.


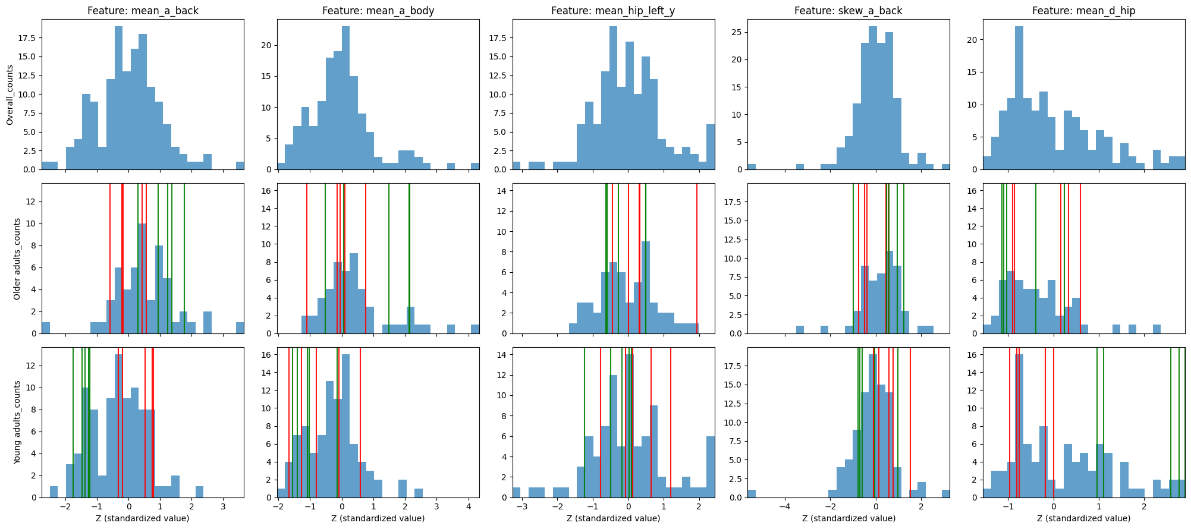
 **Figure S9:** Distribution of features frequently selected for age discrimination.

**|** Cases where models performed well **|** Cases where models did not perform well

Figure S9 Legend: For each feature, the top row represents the overall distribution, the middle row represents the distribution in participants age < 65 years old, bottom row represents the distribution in participants age >= 65 years old. The green vertical dash lines represent 5 cases where they were well classified by the models in each specific age group, while red lines represent 5 cases where they were poorly classified within each specific age group.

**Table S1: Summary and formula of distance features**

| **Feature name** | **Axis** | **Key points** | **Formula** |
| --- | --- | --- | --- |
| d_ankle | $X$ | 28, 29 | $\left\vert X_{28}-X_{29} \right\vert$ |
| d_shoulder | $X$ | 12, 13 | $\left\vert X_{12}-X_{13} \right\vert$ |
| d_hip | $X$ | 24, 25 | $\left\vert X_{24}-X_{25} \right\vert$ |
| d_knee | $X$ | 26, 27 | $\left\vert X_{26}-X_{27} \right\vert$ |
| height_y | $Y$ | highest point, lowest point | $\max(Y)-\min(Y)$ |
| hand_left_y | $Y$ | 16, lowest point | $Y_{16}-\min(Y)$ |
| hand_right_y | $Y$ | 17, lowest point | $Y_{17}-\min(Y)$ |
| shoulder_left_y | $Y$ | 12, lowest point | $Y_{12}-\min(Y)$ |
| shoulder_right_y | $Y$ | 13, lowest point | $Y_{13}-\min(Y)$ |
| ankle_left_y | $Y$ | 28, lowest point | $Y_{28}-\min(Y)$ |
| ankle_right_y | $Y$ | 29, lowest point | $Y_{29}-\min(Y)$ |
| hip_left_y | $Y$ | 24, lowest point | $Y_{24}-\min(Y)$ |
| hip_right_y | $Y$ | 25, lowest point | $Y_{25}-\min(Y)$ |
| knee_left_y | $Y$ | 26, lowest point | $Y_{26}-\min(Y)$ |
| knee_right_y | $Y$ | 27, lowest point | $Y_{27}-\min(Y)$ |

**Table S2: Summary and formula of Angel features**

| **Feature name** | **Key points** | **Formula** |
| --- | --- | --- |
| a_knee_l | 24, 26, 28 | $\angle P_{24}P_{26}P_{28}$ |
| a_knee_r | 25, 27, 29 | $\angle P_{25}P_{27}P_{29}$ |
| a_hip_l | 12, 24, 26 | $\angle P_{12}P_{24}P_{26}$ |
| a_hip_r | 13, 25, 27 | $\angle P_{13}P_{25}P_{27}$ |
| a_ankle_l | 26, 28, 32 | $\angle P_{26}P_{28}P_{32}$ |
| a_ankle_r | 27, 29, 33 | $\angle P_{27}P_{29}P_{33}$ |
| a_top_arm_l | 24, 12, 14 | $\angle P_{24}P_{12}P_{14}$ |
| a_top_arm_r | 25, 13, 15 | $\angle P_{25}P_{13}P_{15}$ |
| a_lower_arm_l | 12, 14, 16 | $\angle P_{12}P_{14}P_{16}$ |
| a_lower_arm_r | 13, 15, 17 | $\angle P_{13}P_{15}P_{17}$ |
| a_body | 12,13,24,25 | Angle between [straight line of (middle point between key point 12 and 13) and (middle point between key point 24, 25)] and [horizontal line] |
| a_back | 8,13,14 | Angle between [key point 8 and (middle point between key point 13, 14)] and [horizontal line] |

**Table S3: Model hyperparameter specifications**

| **Model** | **Prespecified parameters** |
| --- | --- |
| Elastic-net Logistic Regression | Elastic-net mixing parameter (l1 ratio): 0.5  Regularization strength (C): 1.0  Solver: “SAGA”  Maximum iterations: 50,000  Class imbalance: “Class weighting” |
| Histogram-based Gradient Boosting | Maximum tree depth: 2  Minimum samples per leaf: 10  Learning rate: 0.05  Number of boosting iterations: 300 |

**Table S4: Qualitative assessment of walking video among worst performing individuals**

| **Classification** | **Data source** | **General appearance** | **Pose-estimation** | **Clothing** |
| --- | --- | --- | --- | --- |
| Sex | Thailand | Environment appears consistent in all CMU videos | Some jitter as legs pass each other | Trousers |
| Sex | Thailand | Environment appears consistent in all CMU videos | Mostly smooth, some jitter as legs pass each other | Trousers |
| Sex | India | Consistent with other India videos | Jitter in legs | Men’s skirt |
| Sex | India | Consistent with other India videos | Jitter in legs | Men’s skirt |
| Sex | India | Consistent with other India videos | Estimation is mostly smooth | Men’s skirt |
| Age | Thailand | Environment appears consistent in all CMU videos | Estimation is mostly smooth | Trousers |
| Age | India | Camera seems to be pointed more toward the ground than in other videos | Slight jitter in the legs. Bent angle in the knees seems more pronounced in still image than video | Wrapped garment |
| Age | India | Camera seems to be pointed more toward the ground than in other videos | One arm is very bent, the other is not very visible by camera so has some jitter | Trousers |
| Age | India | Camera angle appears to be slightly different in this video | Estimation is mostly smooth | Trousers |
| Age | India | Consistent with other India videos | Estimation is mostly smooth | Men’s skirt |

**Table S5: Qualitative assessment of walking video among best performing individuals**

| **Classification** | **Data source** | **General appearance** | **Pose-estimation** | **Clothing** |
| --- | --- | --- | --- | --- |
| Sex | India | Consistent with other India videos | Some slight jitter as legs pass each other | Wrapped garment but lower parts of legs exposed |
| Sex | India | Consistent with other India videos | Good estimation, though some points disappear for few frames | Wrapped garment |
| Sex | Thailand | Environment appears consistent in all CMU videos | Estimation is smooth | Work dress with lower parts of legs exposed |
| Sex | India | Consistent with other India videos | Estimation is mostly smooth in upper body. Jitter in lower body | Wrapped garment |
| Sex | Thailand | Environment appears consistent in all CMU videos | Participant walks with arms more firmly at their side, so arm facing away from the camera is not visible.  Estimation is mostly smooth | Wearing skirt, lower parts of legs exposed |
| Age | India | Consistent with other India videos | Estimation is mostly smooth, though points seem to disappear for one frame | Wrapped garment |
| Age | India | Consistent with other India videos | Video is slightly choppy/pose estimation not as fluid | Wrapped garment |
| Age | India | Consistent with other India videos | Estimation is mostly smooth | Wrapped garment |
| Age | India | Consistent with other India videos | Estimation is mostly smooth, though points seem to disappear for one frame | Wrapped garment |
| Age | India | Consistent with other India videos | Estimation is mostly smooth in upper body. Jitter in lower body | Wrapped garment |
